# Biopsy-Guided Pathological Response Assessment in Breast Cancer is Insufficient: Additional Pathology Findings of the MICRA Trial

**DOI:** 10.1245/s10434-023-13476-6

**Published:** 2023-04-18

**Authors:** Annemiek K. E. van Hemert, Frederieke H. van Duijnhoven, Ariane A. van Loevezijn, Claudette E. Loo, Terry Wiersma, Emilie J. Groen, Marie-Jeanne T. F. D. Vrancken Peeters

**Affiliations:** 1 Department of Surgical Oncology, Netherlands Cancer Institute – Antoni van Leeuwenhoek, Amsterdam, The Netherlands; 2 Department of Radiation Oncology, Netherlands Cancer Institute – Antoni van Leeuwenhoek, Amsterdam, The Netherlands; 3 Department of Radiology, Netherlands Cancer Institute – Antoni van Leeuwenhoek, Amsterdam, The Netherlands; 4 Department of Pathology, Netherlands Cancer Institute – Antoni van Leeuwenhoek, Amsterdam, The Netherlands; 5 Department of Surgery, Amsterdam University Medical Center, Amsterdam, The Netherlands

## Abstract

**Background:**

Neoadjuvant systemic treatment (NST) leads to pathologic complete response (pCR) in 10–89% of breast cancer patients depending on subtype. The added value of surgery is uncertain in patients who reach pCR; however, current imaging and biopsy techniques aiming to predict pCR are not accurate enough. This study aims to quantify the residual disease remaining after NST in patients with a favorable response on MRI and residual disease missed with biopsies.

**Methods:**

In the MICRA trial, patients with a favorable response to NST on MRI underwent ultrasound-guided post-NST 14G biopsies followed by surgery. We analyzed pathology reports of the biopsies and the surgical specimens. Primary outcome was the extent of residual invasive disease among molecular subtypes, and secondary outcome was the extent of missed residual invasive disease.

**Results:**

We included 167 patients. Surgical specimen showed residual invasive disease in 69 (41%) patients. The median size of residual invasive disease was 18 mm (interquartile range [IQR] 12–30) in hormone receptor-positive (HR+)/human epidermal growth factor receptor 2-negative (HER2−) patients, 8 mm (IQR 3–15) in HR+/HER2-positive (HER2+) patients, 4 mm (IQR 2–9) in HR-negative (HR−)/HER2+ patients, and 5 mm (IQR 2–11) in triple-negative (TN) patients. Residual invasive disease was missed in all subtypes varying from 4 to 7 mm.

**Conclusion:**

Although the extent of residual invasive disease is small in TN and HER2+ subtypes, substantial residual invasive disease is left behind in all subtypes with 14G biopsies. This may hamper local control and limits adjuvant systemic treatment options. Therefore, surgical excision remains obligatory until accuracy of imaging and biopsy techniques improve.

Neoadjuvant systemic treatment (NST) is increasingly being applied as part of tailored breast cancer treatment.^1^ One of the advantages of NST over adjuvant systemic treatment is tumor downsizing, resulting in an increase in breast-conserving therapy rates without compromising local control or overall survival (OS).^2^ Breast cancer subtype is the most predictive factor for the extent to which tumor downsizing occurs. Pathologic complete response (pCR) rates are highest in human epidermal growth factor receptor 2-positive (HER2+) and triple negative (TN) subtypes (40–89%), whereas lower rates are observed in hormone receptor-positive (HR+)/HER2-negative (HER2−) breast cancer (10–15%).^3–11^ As it is questionable whether surgical resection contributes to local control in patients with pCR, less invasive techniques than surgery to assess pCR in patients who underwent NST would be preferable. Since current imaging modalities such as ultrasound, magnetic resonance imaging (MRI), and F18-fluorodeoxyglucose-positron emission tomography/computed tomography (FDG-PET/CT) scan cannot accurately assess pCR,^12^ several international trials aiming to detect pCR using biopsies were initiated. Results of five studies, including the MICRA (Minimal Invasive Complete Response Assessment) trial, were recently published.^13–17^ All five studies differed in radiologic response assessment and biopsy technique but none of these studies achieved a false negative rate (FNR) <10% for the entire study population. The RESPONDER trial (NCT02948764) included 398 patients with a radiologic complete response (rCR) or partial response (rPR) based on mammography, ultrasound or MRI and reported an FNR of 17.8% of 7–10G vacuum-assisted biopsies (VAB) performed with ultrasound or mammography guidance.^15^ Basik et al. reported an FNR of 50% when using 8–11G stereotactic core needle biopsies in the NRG-BR005 trial (NCT03188393) where 98 patients with rCR or near rCR where included. In this trial, response was assessed by mammography (mass ≤1 cm and no malignant microcalcifications), ultrasound (mass ≤2 cm), or MRI (no mass with rapid rise or washout kinetics).^16^ A multi-institutional pooled analysis by Tasoulis et al. including 166 patients with an rCR or rPR on mammography, ultrasound or MRI showed an FNR of 18.7% when 7–14G VAB (*n* = 143) or core-cut biopsies (*n* = 23) were used.^13^ A recent pilot study including 20 patients with an rCR on MRI reported a FNR of 25% of MRI-guided 9G biopsies.^17^

In contrast to these trials, in the MICRA trial, a dynamic contrast-enhanced (DCE)-MRI was mandatory to select patients since DCE-MRI is more accurate in detecting residual disease than mammography or ultrasound.^12^ The FNR (for residual disease) of the ultrasound-guided 14G biopsies in this trial was significantly higher in patients with an rCR on MRI than in patients with an rPR (47% vs. 13%).^14^

Two important studies, the CREATE-X and KATHERINE trials, emphasize the importance of detecting any residual invasive disease in patients with TN and HER2+ breast cancer, respectively, showing that OS can be improved with the administration of additional adjuvant chemotherapy.^18,19^ Therefore, the aim of the present pathology substudy was to quantify the residual disease post-NST and the residual disease missed with biopsies for the different subtypes of breast cancer.

## Methods

### Design/Participants

In this study, the pathology results of biopsies obtained after NST and of the surgical specimen of 167 patients participating in the MICRA trial (NTR6120) were examined and analyzed. The MICRA trial was a prospective single-arm study conducted in three Dutch hospitals.^14^ Eligible patients were women >18 years of age diagnosed with early-stage breast cancer, with an rCR or rPR on DCE-MRI at the end of NST who underwent ultrasound-guided 14G biopsies prior to breast surgery. Definition of an rCR was a complete absence of pathologic contrast enhancement in the original tumor area. rPR was defined according to the Response Evaluation Criteria in Solid Tumors (RECIST) version 1.1 criteria as 0.1–2.0 cm contrast enhancement and ≥30% decrease in tumor size.^20,21^ Breast radiologists obtained the ultrasound-guided 14G biopsies around a pre-NST placed marker in the operating room prior to surgery. Exclusion criteria were histopathologic confirmed ductal carcinoma in situ (DCIS) before the start of NST and a history of ipsilateral breast cancer and/or radiotherapy. Specialized breast pathologists analyzed biopsies and surgical specimen.

### Outcomes

Baseline patient and tumor characteristics were reported by molecular subtype as determined on the pre-NST biopsy. These included age at diagnosis, clinical stage of the tumor (determined with MRI) and lymph nodes (determined with ultrasound and PET/CT), histological tumor subtype, Bloom–Richardson grade, Ki-67 (determined on the pre-NST biopsy), tumor size and multifocality on DCE-MRI, presence of non-mass enhancement on DCE-MRI, presence of microcalcifications on mammography, and radiologic response on MRI. Ki-67 was categorized as ≤5%, between 6% and 29% and ≥30%. Radiologic response in MRI was dichotomized in rPR and rCR.

Post-NST biopsies containing residual tumor cells or signs of the former tumor bed were considered histopathological representative. Post-NST biopsies containing small non-assessable tissue were considered non-representative, and biopsies containing normal breast fatty or connective tissue were considered as unknown.^21^

The pathologic response was described according to the European Society of Breast Cancer Specialists (EUSOMA) 2 criteria. A pCR was defined as the absence of invasive and in situ carcinoma in the surgical specimen, irrespective of nodal status (ypT0Nany). The extent of residual invasive disease in the post-NST biopsy material as well as in the surgical specimen was described by the maximum diameter of the area of residual invasive disease as described in the microscopic report. Residual invasive disease was also classified into three groups based on size: ≤1 mm, larger than 1 mm and <10 mm and ≥10 mm.

### Statistical Analysis

Differences in pathologic response and size of residual invasive disease between subtypes were tested using the Kruskal–Wallis test or Pearson’s Chi-square test. A *p*-value <0.05 was considered statistically significant for comparisons between groups. All statistical analyses were performed using SPSS software version 27 (IBM Corporation, Armonk, NY, USA).

## Results

### Baseline Characteristics

A total of 167 patients were eligible for the analysis of the pathology results of their post-NST biopsies and surgical specimen. Median age was 49 years (interquartile range [IQR] 42–57). The majority of included patients were diagnosed with T2 breast cancer (*n* = 107, 64%) assessed on MRI. Pre-NST imaging showed multifocal disease in 40 patients (24%), calcifications on mammography in 30 patients (18%), and non-mass enhancement in 28 patients (17%).

Pre-NST biopsies showed invasive carcinoma of no special type in 146 patients (87%), invasive lobular carcinoma in 14 patients (9%), and other special type carcinomas in 7 patients (4%).

Table 1 shows the baseline characteristics by molecular subtype. Furthermore, grade and Ki-67 were assessed on the pre-NST biopsies; half of the HR+ patients (HR+/HER2−: *n* = 23, 54%; HR+/HER2+: *n* = 22, 54%) presented with grade 2 disease, whereas the majority of the HR− patients (HR−/HER2+: *n* = 14, 61%; TN: *n* = 50, 83%) had grade 3 disease. Pre-NST Ki-67 was ≥30% in 65% (*n* = 77) of all patients, with the highest percentages seen in TN breast cancer (*n* = 44, 94%).

**Table 1:** Baseline characteristics by subtype [*n =* 167]

	HR+/HER2− [*n *= 43 (26%)]	HR+/HER2+ [*n *= 41 (25%)]	HR−/HER2+ [*n *= 23 (14%)]	TN [*n *= 60 (36%)]
Age at surgery	50 (42–57)	49 (44–56)	56 (47–65)	47 (39–53)
*T stage at diagnosis*				
T1	6 (14)	9 (22)	5 (22)	16 (27)
T2	33 (77)	23 (56)	14 (61)	37 (62)
T3	4 (9)	8 (20)	4 (17)	7 (12)
T4	1 (0)	1 (2)	0 (0)	0 (0)
*N stage at diagnosis*				
N0	13 (30)	20 (49)	14 (61)	36 (60)
N1	20 (47)	16 (39)	4 (17)	(17)
N2	7 (16)	1 (2)	3 (13)	10 (8)
N3	3 (7)	4 (10)	2 (9)	5 15)
*Histology*				
No special type	32 (74)	35 (86)	22 (96)	57 (95)
Lobular	7 (16)	5 (12)	1 (4)	1 (2)
Other	4 (9)	1 (2)	0 (0)	2 (3)
*Grade*				
1	5 (12)	1 (2)	1 (4)	0 (0)
2	23 (54)	22 (54)	3 (13)	8 (13)
3	14 (33)	17 (42)	14 (61)	50 (83)
Unknown	1 (2)	1 (2)	5 (22)	2 (3)
*Ki-67 pre-NST, %*	^a^	^b^	^c^	^d^
≤5	5 (14)	1 (5)	0 (0)	0 (0)
6–29	19 (53)	11 (50)	3 (21)	3 (6)
≥30	12 (33)	10 (45)	11 (79)	44 (94)
*Imaging features*				
Multifocal	11 (26)	17 (42)	4 (17)	8 (13)
Non-mass	15 (35)	7 (17)	2 (9)	4 (7)
Calcifications	12 (28)	10 (24)	3 (13)	5 (8)
*Radiologic response (MRI)*				
rCR	32 (74)	36 (88)	21 (91)	47 (78)
rPR	11 (26)	5 (12)	2 (9)	13 (22)

Data are expressed as *n* (%) or median (IQR)

^a^7 unknown values, ^b^19 unknown values, ^c^9 unknown values, ^d^13 unknown values

*HR* Hormone receptor, *HER2* Human epidermal growth factor receptor 2, *IQR* Interquartile range, *MRI* Magnetic resonance imaging, *NST* Neoadjuvant systemic therapy, *rCR* Radiologic complete response, *rPR* Radiologic partial response, *TN* Triple negative

After NST, most patients (81%, *n* = 136) had an rCR on MRI and 19% of patients (*n* = 31) had an rPR.

### Pathology Results of the Surgical Specimen of All Patients

In total, 89 patients (53%) had a pCR of the breast after NST. The pCR rates were highest in patients with Ki-67 ≥30% (pCR rate 61%) and lowest in patients with Ki-67 ≤5% (pCR rate 17%).

Nine patients (5%) had solely residual DCIS and 69 patients (41%) had residual invasive disease in the breast. Pathologic response and size of invasive residual disease did not vary across clinical tumor status (*p*-value 0.698 and 0.678, respectively).

As shown in Table 2; residual invasive disease after NST was most often seen in patients with HR+/HER2− subtype of breast cancer (*n* = 31, 72%) and patients with this subtype of breast cancer also had the largest size of residual invasive disease (18 mm, IQR 12–30) .

**Table 2:** Pathology results of surgical specimen per subtype [*n* = 167]

	HR+/HER2− [*n* = 43]	HR+/HER2+ [*n* = 41]	HR−/HER2+ [*n* = 23]	TN [*n* = 60]	*p*-Value^a^
*Pathologic response*					< 0.001
Pathologic complete response	9 (21)	24 (59)	16 (70)	40 (67)	
No residual invasive but DCIS	3 (7)	1 (2)	2 (9)	2(3)	
Minimal residual disease, <10%	11 (26)	14 (34)	3 (13)	11 (18)	
10–50% of tumor remaining	14 (33)	2 (5)	2 (9)	5 (8)	
>50% of tumor remaining	6 (14)	0 (0)	0 (0)	1 (2)	
Only LVI present	0 (0)	0 (0)	0 (0)	1 (2)	
Invasive residual disease	31 (72)	16 (39)	5 (22)	17 (28)	< 0.001
Size of invasive residual disease, mm	18 (12–30)	8 (3–15)	4 (2–9)	5 (2–11)	< 0.001
*Residual invasive disease, mm*					< 0.001
≤1	1 (2)	2 (5)	1 (4)	4 (7)	
>1–10	6 (14)	10 (24)	4 (17)	9 (15)	
>10	24 (56)	4 (10)	0 (0)	4 (7)	

Data are expressed as *n* (%) or median (IQR)

*HR* Hormone receptor, *IQR* Interquartile range, *HER2* Human epidermal growth factor receptor 2, *DCIS* Ductal carcinoma in situ, *LVI*Lymphovascular invasion, *TN* Triple negative

^a^Kruskal–Wallis test/Pearson’s Chi-square test

Patients with HR−/HER2+ subtype had the lowest rate of residual invasive disease (*n* = 5, 22%).

### Accuracy of the Biopsies

Table 3 shows the pathology results of the post-NST biopsies. In the majority of patients (*n* = 161, 96%) six or more biopsies were obtained, and in 86% of the patients (*n* = 144) eight biopsies were obtained. The biopsies were representative in 156 patients (93%). In 65 of the 69 patients with residual invasive disease, the biopsies were representative. In only 58% of these 65 patients (*n* = 38), this residual invasive disease was detected in the biopsies, thus in the remaining 27 patients (42%), the residual invasive disease was missed.

**Table 3:** Pathology results of post-NST biopsies per subtype [*n* = 167]

	HR+/HER2− [*n* = 43]	HR+/HER2+ [*n* = 41]	HR−/HER2+ [*n* = 23]	TN [*n* = 60]
*Biopsies representative*				
No (*n* = 11)	1 (2)	3 (7)	3 (13)	4 (7)
Yes (*n* = 156)	42 (98)	38 (93)	20 (87)	56 (93)
*Residual disease in the representative biopsies*				
No disease	17 (40)	5 (66)	17 (85)	47 (84)
Any disease	25 (60)	13 (34)	3 (15)	9 (16)
Invasive disease	22	9	0	7
In situ disease	7	6	3	2
True positive^a^	25	7	0	6
False negative^a^				
False negative, invasive disease (FNR)	(26)	6 (38)	5 (100)	8 (47)
False negative, in situ disease	8	3	1	5

Data are expressed as *n* (%) or median

^a^Only representative biopsies were included in the analyses. In situ disease includes DCIS, LCIS, and LVI

*HR* Hormone receptor, *HER2* Human epidermal growth factor receptor 2, *DCIS* Ductal carcinoma in situ, *LCIS* Lobular carcinoma in situ, *LVI* Lymphovascular invasion, *FNR* False negative rate, *TN* triple negative

The FNR for residual invasive disease of (representative) post-NST biopsies was the highest in the TN subtype and HR−/HER2+ subtype (53% and 100%, respectively). The lowest FNR was found in the HR+/HER2+ subtype and HR+/HER2− subtype (38% and 26%, respectively).

### Pathology Results of Patients with Missed Residual Invasive Disease

Table 4 shows the details of the pathologic response and size of residual invasive disease in patients where residual invasive disease was missed by representative post-NST biopsies. Residual invasive disease is missed in particular in patients with minimal residual disease (<10%, 78%). The largest median size of missed residual invasive disease was found in patients with HR+ breast cancer (7 mm). The median size of missed residual invasive disease in HR−/HER2+ and TN subtypes was 4 mm (IQR 2–9, IQR 1–10). Residual invasive disease of >1 mm was missed in all subtypes (*n* = 21) and residual invasive disease ≥10 mm was missed in patients with HR+/HER2− (*n* = 2) and TN subtypes (*n* = 2).

**Table 4:** Pathology results of the surgical specimen of patients with missed residual invasive disease, by subtype [*n *= 27]

	HR+/HER2− [*n* = 8]	HR+/HER2+ [*n* = 6]	HR−/HER2 + [*n* = 5]	TN [*n* = 8]
*Pathologic response*				
Minimal residual disease, <10%	6	6	3	6
10–50% of tumor remaining	2	0	2	1
>50% of tumor remaining	0	0	0	1
No evidence of response	0	0	0	0
Size of residual invasive disease	7 (3–16)	7 (3–8)	4 (2–9)	4 (1–10)
*Residual invasive disease, mm*				
≤1	1	1	1	2
>1–10	5	5	4	4
≥10	2	0	0	2

Data are expressed as *n* or median (IQR)

Only representative and unknown representative biopsies were included in the analyses

*HR* Hormone receptor, *HER2* Human epidermal growth factor receptor 2, *IQR* Interquartile range, *TN* Triple negative

## Discussion

With the development of tailored NST, the number of patients undergoing BCS after NST has increased, and depending on subtype, 10–89% of patients will have a pCR after NST.^3–11^ The added value of surgery, other than as a diagnostic tool to assess the pathologic response, is uncertain in patients who reach a pCR. However, in TN and HER2+ patients, it is necessary to detect all residual invasive disease since adjuvant systemic treatment improves survival in these patients. Several international trials investigating pCR detection by minimally invasive techniques show high FNR.^13–17^ The current analysis of pathology results of patients included in the MICRA trial provides an overview of residual invasive disease found after NST in patients with a favorable response on MRI after NST.

Although we found that the vast majority (93%) of the post-NST biopsies were representative (meaning that the obtained tissue contained residual tumor cells, signs of the former tumor bed, normal fatty or connective tissue of the breast), FNR rates of the biopsies were high in all subtypes.

The limited residual invasive disease found in the surgical specimen in HER2+ and TN subtypes can explain the high FNR (38–100%), while it is precisely these subtypes where any residual disease should be detected in order not to withhold adjuvant therapy from these patients.^14^ In HER2+ and TN patients, biopsies relatively often showed false negative results, leaving 4–7 mm of residual invasive disease behind when solely relying on the biopsies.

In the MICRA study, we relied on the most optimal response monitoring strategy using MRI imaging^22,23^ followed by ultrasound-guided 14G biopsies post-NST. In comparable studies, less accurate imaging modalities for response monitoring were used in combination with larger tissue samples such as VAB, resulting in lower FNRs.^13,15,16^ However, the question remains whether larger biopsies are able to detect residual disease with a desirable FNR of 0% in HER2+ and TN patients.

The results of the present study show that only limited residual invasive disease is present in patients with a favorable response on MRI. Although the absolute benefit of adjuvant systemic therapy in patients with minimal residual invasive disease is likely to be lower, this has not yet been proven. Two recent practice-changing studies, the CREATE-x and KATHERINE trials, have emphasized the importance of detecting any residual invasive disease in HER2+ and TN breast cancer patients after NST, since adjuvant chemotherapy improves survival in these patients.^18,19^ In addition to the KATHERINE trial in which adjuvant trastuzumab emtansine (T-DM1) proved survival benefit in HER2+ patients with residual disease,^19^ the ExteNET trial showed significant improved survival in HR+/HER2+ patients with (any) residual disease in the breast or axilla receiving adjuvant neratinib.^24^ Results of an ongoing trial on trastuzumab deruxtecan (T-DXd) versus T-DM1 in high-risk HER2+ patients with (any) residual invasive disease are yet to follow.^25^

Among ongoing trials on the added value of adjuvant systemic therapy for TN breast cancer patients with residual invasive, definition of (relevant) residual disease varies from any, >1 mm, or ≥10 mm.^26^ In our cohort, of the 27 patients in whom residual invasive disease was missed by representative biopsies, 78% (*n* = 21) had residual invasive disease larger than 1 mm in the surgical specimen. In four patients, including TN breast cancer patients, residual invasive disease larger than 10 mm was found.

All HR+ patients treated in neoadjuvant setting are treated with adjuvant hormonal therapy regardless of the response to NST, and currently no adjuvant chemotherapy is indicated for these patients. Thus, in the subgroup of HR+/HER2− patients, the residual disease that is potentially missed when omitting surgery after a false negative biopsy could potentially lead to a higher recurrence rate. Into what extent the recurrence rate will increase and how this influences survival rates is a topic for further research.

As mentioned earlier, the use of larger VAB could result in a lower FNR, as shown by Tasoulis et al.^27^, and this technique is already used to omit breast surgery in patients with VAB-confirmed pCR.^28^ In a multicenter, single-arm trial, breast cancer patients with TN and HER2+ subtype had a minimum of 12 vacuum-assisted 9G core biopsies, after which breast surgery was omitted in 31 patients with a pCR as assessed by these biopsies.^28^ Median follow-up of 26 months showed no ipsilateral breast cancer recurrences in patients where breast surgery was omitted. With a mean of 15 vacuum-assisted 9G core biopsies obtained in each patient, one may question whether this can be considered as a minimally invasive technique. In addition, eight patients with nodal disease and a biopsy-confirmed breast pCR in whom breast surgery was omitted still received targeted axillary dissection in the operating room.^28^

In our attempt to de-escalate locoregional treatment in favorable responders to NST, another option would be to select a group of patients where radiotherapy could be omitted. Omission of radiotherapy was already investigated in older breast cancer patients (>65 years of age) following primary surgical treatment. This resulted in a higher local recurrence rate in the experimental arm (4.1% vs. 1.3%), but no difference in OS^29^ was observed. Omission of radiotherapy in breast cancer patients achieving pCR after NST is currently being investigated in the DESCARTES trial (NCT05416164), in which adjuvant radiotherapy is omitted in cT1-2N0 breast cancer patients (all ages, all subtypes) who achieve pCR of the breast (surgical lumpectomy) and lymph node[s] (sentinel node) after NST (NCT05416164).

## Conclusion

To enable patient-tailored adjuvant treatment depending on the subtype in the residual disease, it is of importance to find any residual disease in HER2+ and TN subtypes. Accurate assessment of pathologic response using the currently available minimally invasive techniques, including (small) biopsies, does not yet seem possible. Therefore, reconsideration of the omission of surgery is necessary and alternative de-escalation strategies such as de-escalation of radiotherapy in pCR patients must be investigated.
